# Purkinje de-networking for refractory short-coupled premature ventricular contraction–triggered ventricular fibrillation in dextrocardia with situs inversus

**DOI:** 10.1016/j.hrcr.2026.06.014

**Published:** 2026-06-17

**Authors:** Shabnam Madadi, Morvarid Mirzaeian Khamsheh, Maryam Tahamtan, Farzad Kamali, Seyed Shahin Eftekhari

**Affiliations:** 1 Cardiac Electrophysiology Research Center, Rajaie Cardiovascular Institute, Tehran, Iran; 2 Cardiovascular Research Center, Rajaie Cardiovascular Institute, Tehran, Iran

**Keywords:** Purkinje fibers, Ventricular fibrillation, Short-coupled premature ventricular contractions, Dextrocardia, Catheter ablation, Purkinje de-networking

**Key Teaching Points**

• Short-coupled premature ventricular complexes arising from the Purkinje system are a malignant trigger of polymorphic ventricular tachycardia and ventricular fibrillation, even in structurally normal hearts. Recognition of a fixed short coupling interval and catecholamine sensitivity is critical for diagnosis.
• Transient QT prolongation after ventricular fibrillation or resuscitation should not automatically be interpreted as congenital long QT syndrome. Careful clinical correlation is essential before attributing arrhythmia mechanisms to genetic findings.
• Purkinje de-networking is an effective substrate modification strategy for refractory Purkinje-triggered ventricular fibrillation, particularly when spontaneous ectopy is suppressed during deep sedation.
• A combined antegrade transseptal and retrograde aortic approach can facilitate comprehensive mapping and ablation in complex anatomical settings such as dextrocardia with situs inversus.
• Genetic variants, including those in *AKAP9*, may act as modifiers of repolarization reserve rather than primary disease-causing substrates and should therefore be interpreted cautiously within the overall clinical context.

## Introduction

Dextrocardia with situs inversus occurs in approximately 1–6 per 10,000 individuals and poses important technical challenges during cardiac procedures, including catheter ablation.^1^, ^2^, ^3^ Idiopathic ventricular fibrillation (VF) mediated by Purkinje ectopy is increasingly recognized as a distinct clinical entity,^4^ and short-coupled premature ventricular contractions (SC-PVCs) are established triggers of polymorphic ventricular tachycardia (PMVT) or VF even in structurally normal hearts.^5^^,^^6^ Ablation strategies targeting Purkinje potentials have emerged as effective therapies in this setting.^7^, ^8^, ^9^, ^10^, ^11^

Genetic factors may further modulate susceptibility to malignant ventricular arrhythmias by reducing repolarization reserve. *AKAP9* encodes Yotiao, a regulatory scaffold protein that modulates the slow delayed rectifier potassium current (I_Ks_) through interaction with *KCNQ1*. Variants in *AKAP9* have been associated with dynamic or stress-unmasked QT prolongation, reflecting impaired I_Ks_ augmentation during physiologic or sympathetic activation. Such repolarization vulnerability may increase transmural dispersion and facilitate Purkinje hyperexcitability, potentially lowering the threshold for VF.^12^

This appears to be the first reported case of successful Purkinje de-networking (PDN) combined with focal PVC ablation in a patient with the rare coexistence of dextrocardia, Purkinje-triggered PMVT/VF, and *AKAP9*-related repolarization vulnerability.

## Case presentation

**Figure 1:**
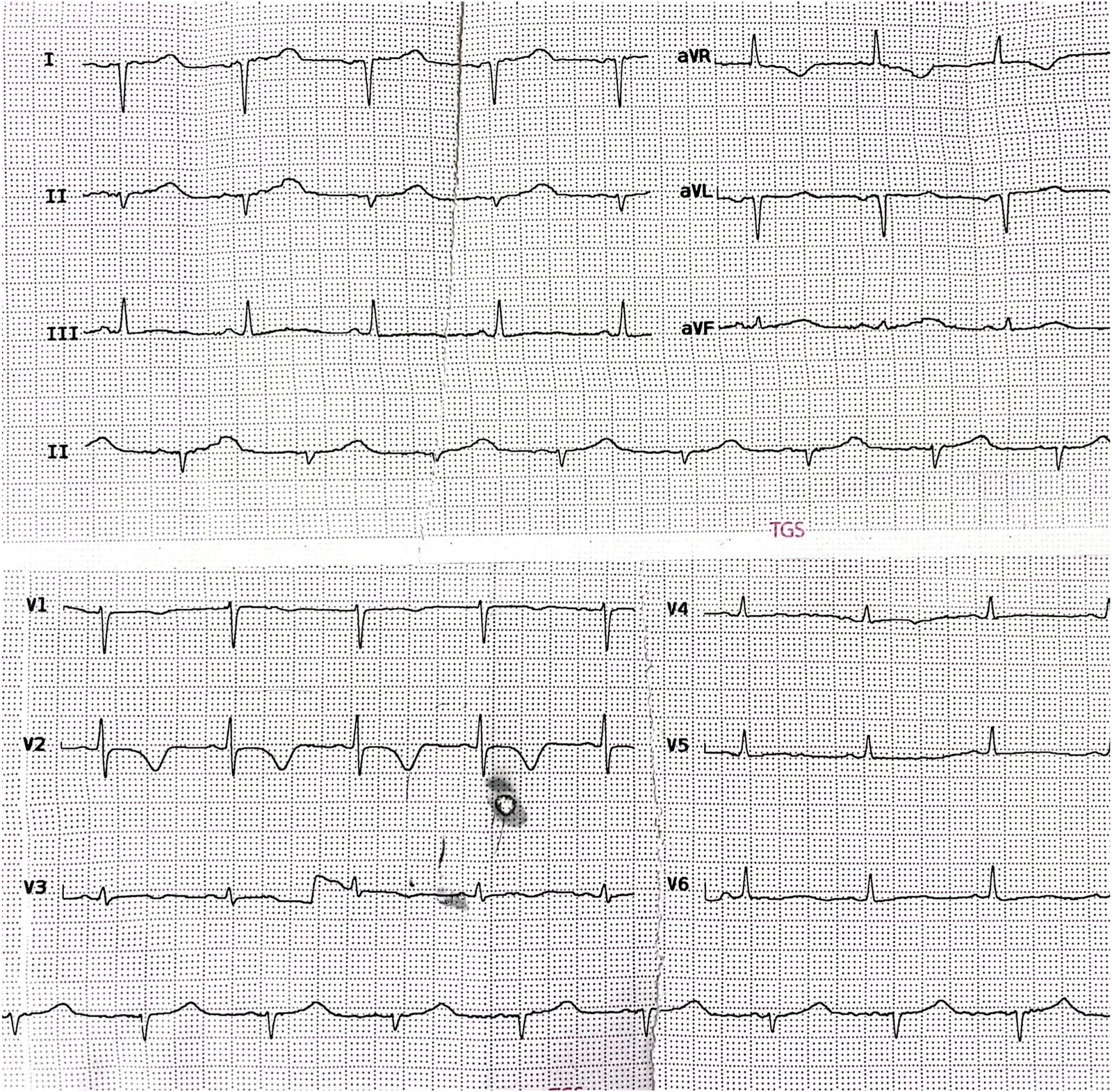
Baseline 12-lead electrocardiogram demonstrating sinus rhythm with right axis deviation and negative P waves in lead I. Note that limb leads are placed as standard and precordial leads are reversed in a mirror-image pattern. The corrected QT interval was measured at approximately 530 ms, with no evidence of T-wave alternans. TGS = tracing gain and speed.

**Figure 2:**
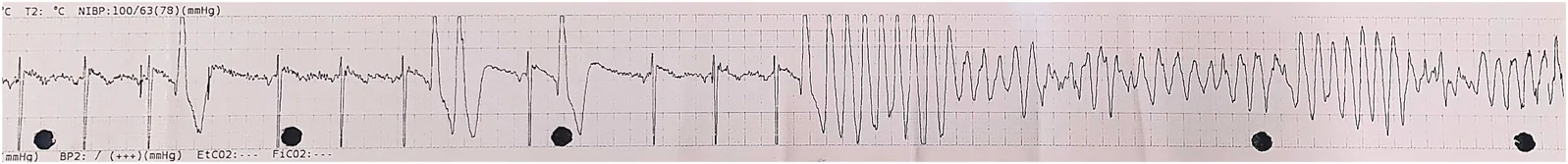
Recorded single-lead electrocardiogram showing an episode of polymorphic ventricular tachycardia initiated by a short-coupled premature ventricular contraction (coupling interval 310 ms).

A 43-year-old woman with a long-standing history of unexplained syncope was transferred to our center after a short run of PMVT was recorded during hospitalization. During transport, she experienced several additional syncopal episodes. On arrival, a 12-lead electrocardiogram showed sinus rhythm with right axis deviation, negative P waves in lead I, and a corrected QT (QTc) interval of 530 ms measured using the Bazett formula (Figure 1). No T-wave alternans was present. Serum electrolytes were normal, and the patient was not taking QT-prolonging medications. Transthoracic echocardiography demonstrated dextrocardia with situs inversus and no structural heart disease. Continuous monitoring revealed frequent episodes of PMVT/VF, each consistently triggered by a short-coupled premature ventricular complex (SC-PVC) with a fixed coupling interval (Figure 2).

Propranolol therapy was initiated and titrated to 40 mg 4 times daily. Mexiletine and intravenous magnesium sulfate were also administered. The patient subsequently underwent bilateral stellate ganglion block, and a dual-chamber implantable cardioverter-defibrillator (ICD) was implanted for secondary prevention.

Genetic testing (whole-exome sequencing) identified 2 closely spaced heterozygous frameshift insertions in AKAP9, NM_005751.5: 7:92097636A>ATT (c.10449_10450insTT; p.Q3484Ffs∗4) and, 7:92097637C>CT (c.10450_10451insT; p.Q3484Lfs∗14).

These variants introduce premature stop codons and result in predicted truncation of Yotiao. Although the laboratory classified these variants as likely pathogenic, the transient nature of the presenting QT prolongation suggested reduced repolarization reserve rather than definitive congenital long QT syndrome. Cascade testing of first-degree relatives is ongoing; initial screening identified an additional heterozygous *AKAP9* variant (NM_005751, exon 18: c.4768_4769insTG; p.M1590fs, chr7:91670063) in 1 family member, without clinical manifestations.

**Figure 3:**
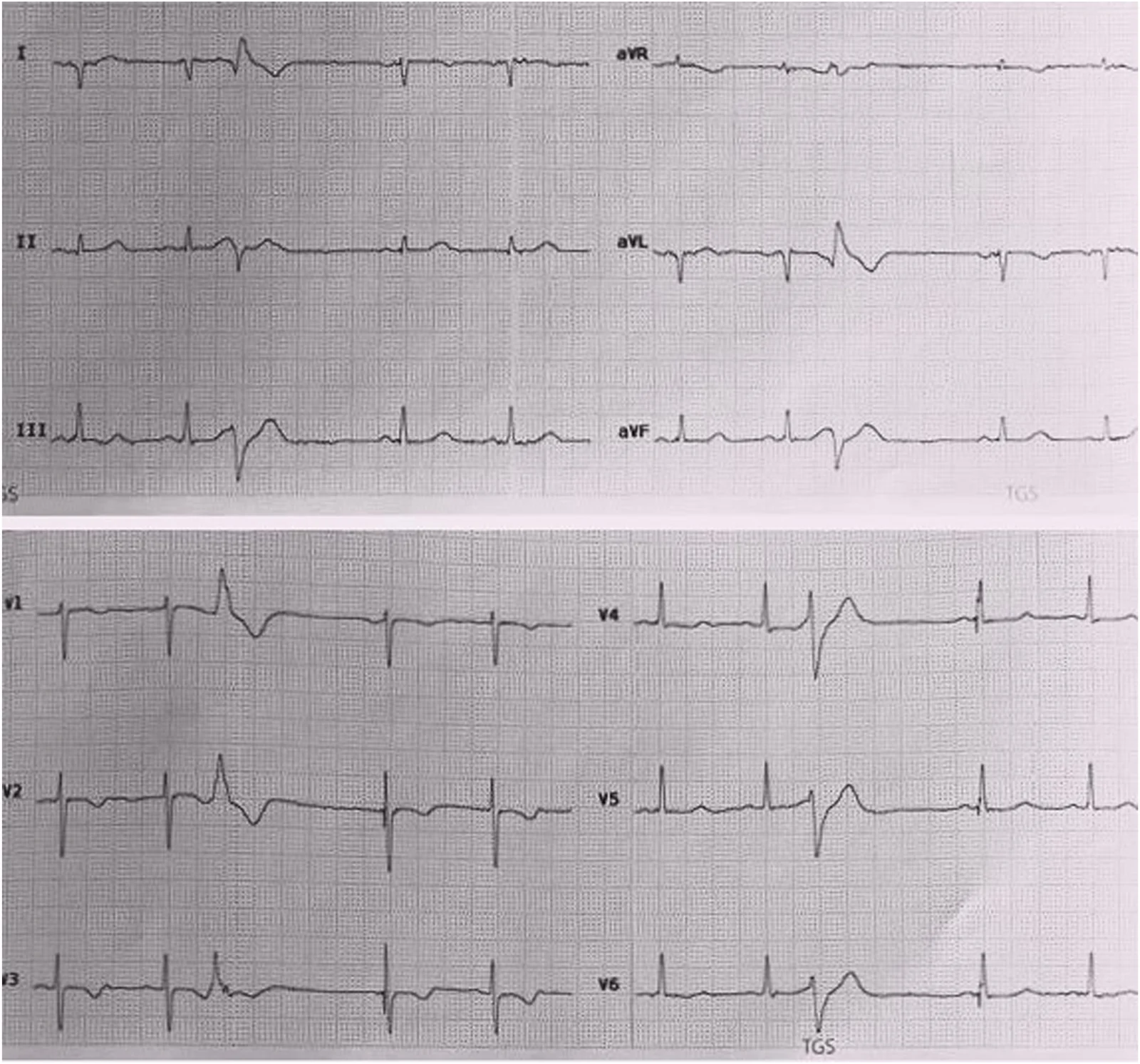
12-Lead electrocardiogram indicating sinus rhythm with short-coupled premature ventricular contractions and a normal corrected QT interval (447 ms). Note that limb leads are placed as standard and precordial leads are reversed in a mirror-image pattern. TGS = tracing gain and speed.

The patient was discharged on propranolol and mexiletine. She remained asymptomatic for several weeks before presenting again with a refractory PMVT/VF storm resulting in 18 appropriate ICD shocks. During this relapse, episodes were again initiated by unifocal SC-PVCs; however, the QTc interval was normal (Figure 3). Intravenous verapamil was added, and deep sedation temporarily suppressed arrhythmias, but PMVT/VF recurred each time sedation was lightened.

The initiating PVCs, despite a QRS duration of 140 ms, were consistent with Purkinje-mediated triggers because of their short coupling interval, catecholamine sensitivity, and suppression by sedation. The PVC morphology showed a right bundle branch block pattern with a superior axis, consistent with reversed precordial orientation and suggesting origin from the posterior Purkinje–papillary muscle region, likely near the left posteroseptal papillary muscle Purkinje–muscle junction.

### Electrophysiology procedure

Given the recurrent Purkinje-triggered VF, the decision was made to perform PDN. Preprocedural contrast-enhanced computed tomography provided detailed delineation of the mirror-image cardiac configuration.

In the electrophysiology laboratory, under general anesthesia and with mechanical circulatory support on standby, bilateral femoral venous access was obtained for right ventricular and coronary sinus catheter placement and for potential transseptal access. A right femoral arterial sheath was inserted for hemodynamic monitoring and for enabling a retrograde aortic approach, if needed.

**Figure 4:**
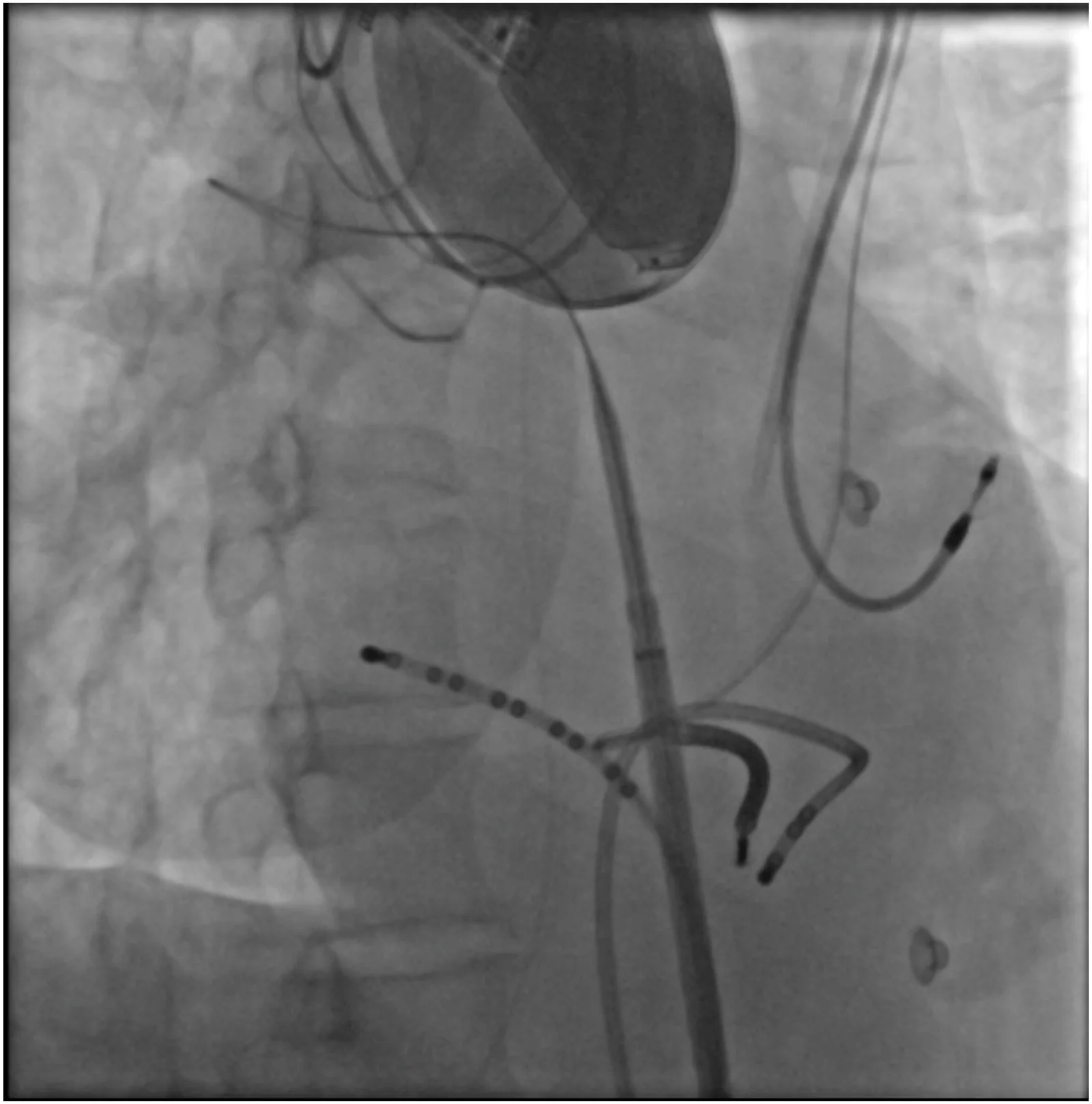
Right anterior oblique view confirming the transseptal puncture by visualization of the guidewire traversing into the pulmonary vein.

A pigtail catheter was advanced into the right atrium for contrast injection to delineate atrial anatomy. Transseptal puncture was performed in the right anterior oblique view using a mid-Brockenbrough transseptal needle (Abbot, St.Paul, MN) through an Agilis NxT steerable sheath (Abbot, St.Paul, MN). Entry into the left atrium was confirmed by contrast injection and visualization of the guidewire advancing into the right upper pulmonary vein (Figure 4).

**Figure 5:**
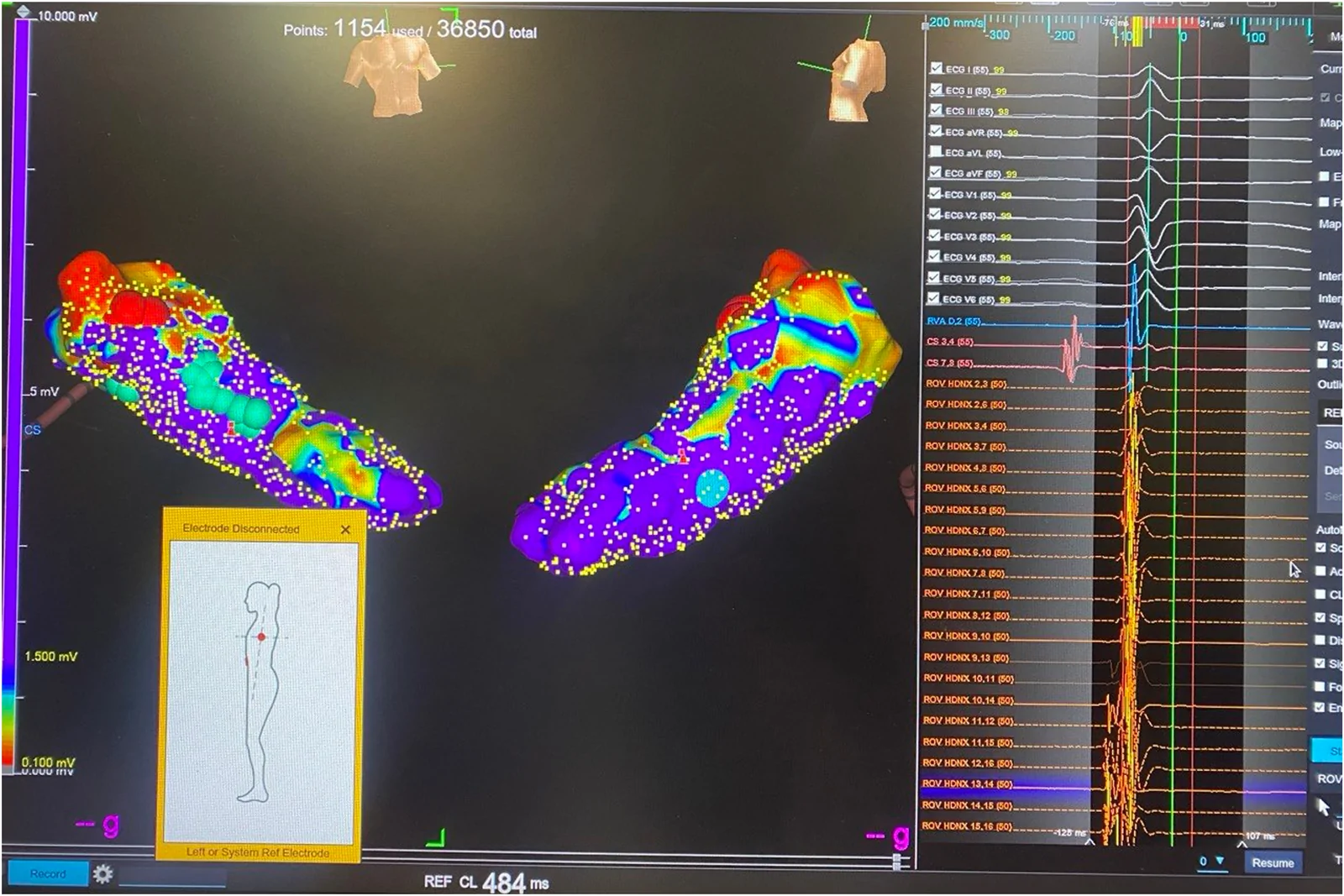
EnSite X 3-dimensional map illustrating Purkinje network and ablation sites.

Electroanatomic mapping was performed using the Advisor HD Grid mapping catheter (Abbott, St.Paul, MN) and the 3-dimensional EnSite X system (Figure 5). Via a transaortic approach, extensive distal Purkinje potentials were annotated across the mid-to-apical anterior and posterior left ventricular endocardium. No PVCs occurred during deep sedation, consistent with catecholamine-sensitive Purkinje triggers.

**Figure 6:**
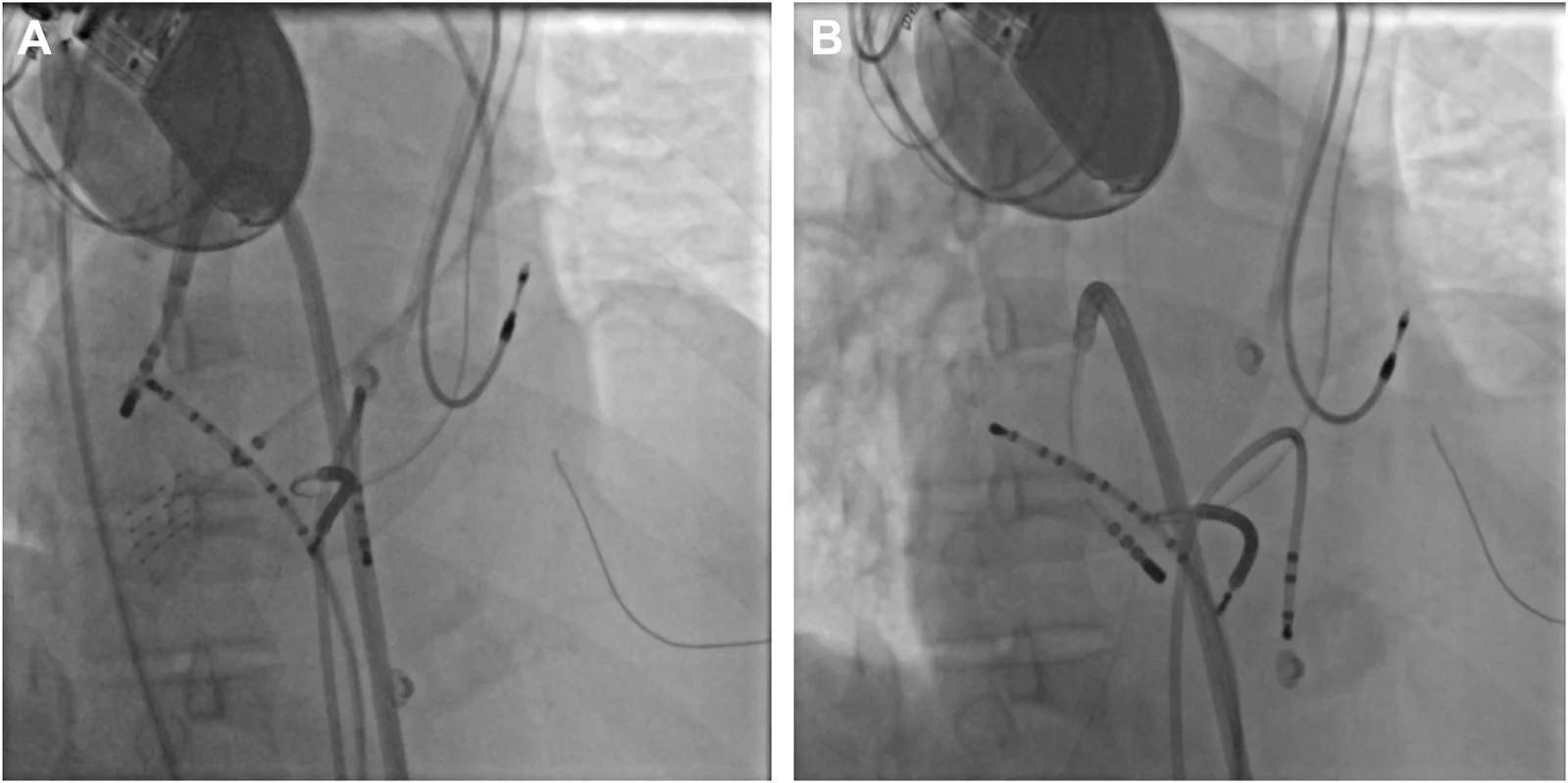
Right anterior oblique views showing the position of the ablation catheter adjacent to distal anterior (A) and posterior (B) left ventricular Purkinje fibers. The coronary sinus and right ventricular catheters are also seen, as well as a mapping catheter in panel A.

**Figure 7:**
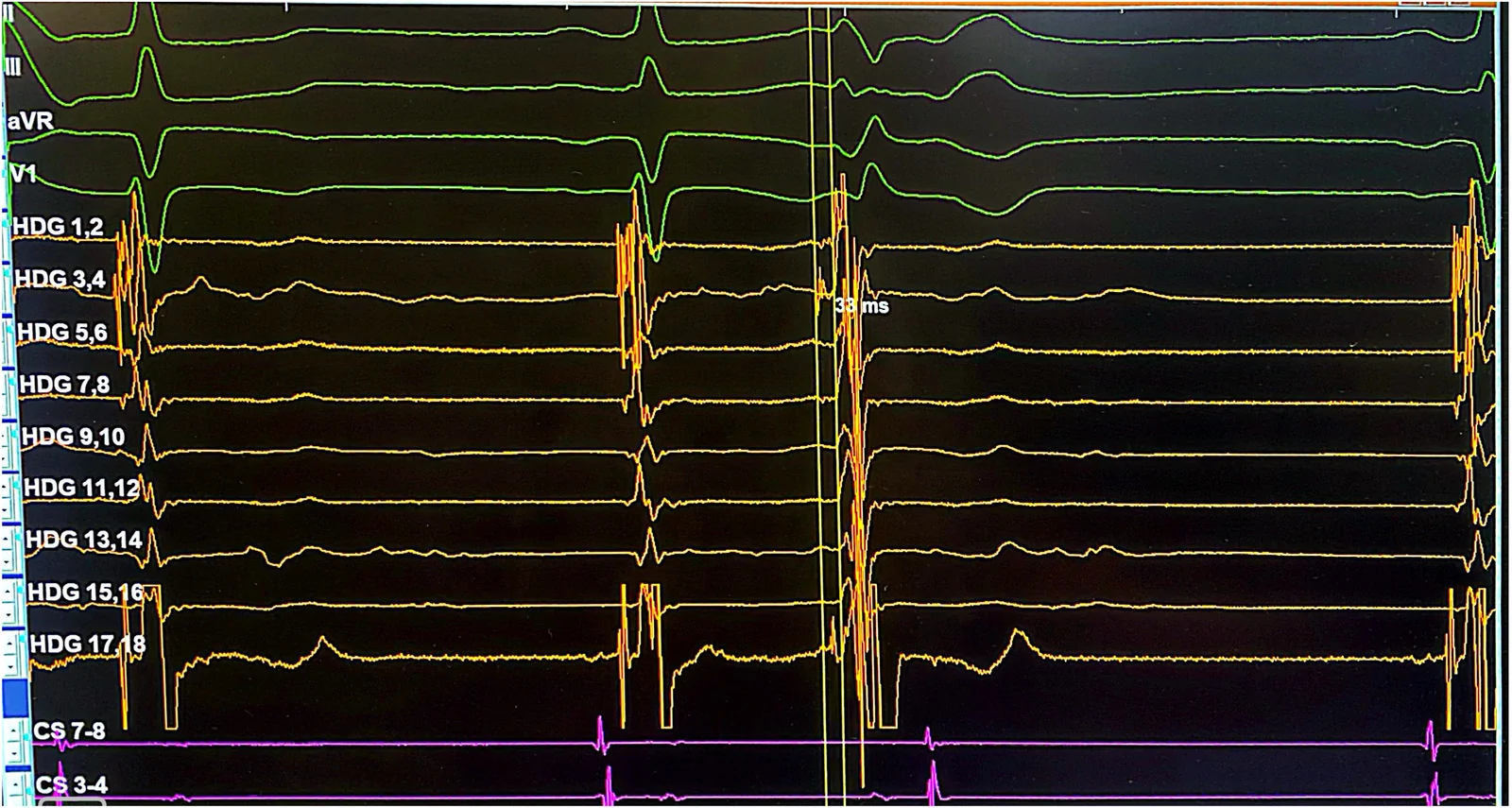
The earliest signal of unifocal Purkinje-related premature ventricular contractions. Image acquired using the CamScanner smartphone application.

Linear radiofrequency ablation was delivered along the distal Purkinje arborization and extended proximally while sparing the left bundle branch and fascicles, completing PDN using a TactiFlex ablation catheter (Abbott) via an antegrade approach (Figure 6A and 6B). Sedation was then gradually reduced, leading to reappearance of the unifocal SC-PVCs. Activation mapping identified the earliest Purkinje potential 33 ms before QRS onset (Figure 7) at the Purkinje–papillary muscle interface of the left posteromedial papillary muscle. Targeted ablation at this site eliminated the PVCs. Upon awakening, programmed ventricular stimulation failed to induce ventricular arrhythmias.

The patient remained free of ventricular arrhythmias and ICD therapies during 6-month follow-up. Propranolol therapy continued, whereas mexiletine was discontinued after ablation.

## Discussion

This case illustrates an uncommon and highly challenging presentation of short-coupled premature ventricular complexes (SC-PVCs) triggering PMVT/VF in the setting of mirror-image dextrocardia and multiple truncating variants in *AKAP9*. The interplay of an anatomic substrate with a hyperexcitable Purkinje network and potentially reduced repolarization reserve provides a plausible framework for understanding this malignant arrhythmia phenotype. The case also highlights the therapeutic usefulness of a combined PDN and focal ablation approach.

### Malignant nature of Purkinje-triggered ventricular arrhythmias

SC-PVC–mediated PMVT/VF is one of the most lethal forms of idiopathic ventricular arrhythmia. Hallmark features, including a fixed short coupling interval, Purkinje potentials preceding PVC onset, catecholamine sensitivity, and rapid degeneration into PMVT/VF,^5^^,^^6^ were all present in our patient group. Earlier literature distinguished SC-PVC–mediated VF from torsades de pointes even when QT prolongation coexisted, leading to the term “pseudo–torsades de pointes.”^13^^,^^14^ More recent work suggests that overlap may occur in certain settings.^15^^,^^16^ In our case, although the QT interval was initially prolonged, its transient nature, the absence of T-wave alternans, and normalization during recurrent VF episodes (QTc interval ∼447 ms) argue against congenital long QT syndrome as the primary mechanism.

Myocarditis was considered as an explanation for transient QT prolongation, but the normal cardiac biomarkers and echocardiography made this unlikely. Cardiac magnetic resonance imaging could not be performed because of hemodynamic instability and lack of wide-band technology. Quinidine, known to suppress SC-PVC–mediated idiopathic VF,^17^^,^^18^ was avoided because of baseline QT prolongation and concern for further repolarization delay.

### Electrophysiological considerations in dextrocardia

Dextrocardia with situs inversus introduced unique technical challenges. All fluoroscopic views were reversed, catheter torque responses were inverted, and papillary muscle orientation was mirrored. Preprocedural computed tomography imaging was essential for understanding the spatial configuration and guiding safe transseptal access. High-density mapping allowed visualization of Purkinje arborization in the reversed anatomy. Deep sedation suppressed spontaneous PVCs, making initial focal mapping unfeasible. PDN was therefore performed first to reduce distal Purkinje excitability and facilitate the reappearance of SC-PVCs once sedation was lightened. This sequential strategy, substrate modification followed by targeted ablation, successfully eliminated the unifocal PVCs and rendered VF noninducible, resulting in complete arrhythmia suppression during follow-up.

### Genetic background and interaction with Purkinje physiology

The patient harbored 2 truncating *AKAP9* variants. *AKAP9* encodes Yotiao, a scaffolding protein involved in symptomatic-dependent augmentation of I_Ks_. Variants in *AKAP9* have been associated with reduced repolarization reserve and dynamic QT prolongation in certain contexts, including adrenergic stimulation and physiological stress.^19^ In our patient, the presence of transient QT prolongation may reflect such repolarization vulnerability, although the available data are insufficient to diagnose congenital long QT syndrome type 11. Importantly, QT interval normalized during recurrent VF episodes, and the genetic findings were not accompanied by a clear family phenotype. Therefore, these variants are best interpreted as possible modifiers that may have contributed to increased susceptibility rather than a primary cause of VF.

Although reduced I_Ks_ reserve has been proposed to influence repolarization dynamics,^12^ direct evidence linking *AKAP9* variants to Purkinje excitability in humans remains limited. Although the coexistence of SC-PVCs and transient QT prolongation in this patient is observed temporally, causality cannot be established. Regardless of the underlying genetic substrate, elimination of Purkinje triggers through PDN and focal ablation was sufficient to suppress VF, underscoring the critical role of trigger elimination in Purkinje-mediated arrhythmias.

Although dextrocardia with situs inversus is not genetically linked to *AKAP9* variants, mirror-image cardiac geometry may alter Purkinje distribution and potentially influence conduction heterogeneity. The combination of an inverted Purkinje network and possible reduction in repolarization reserve may have collectively facilitated the initiation and maintenance of malignant ventricular arrhythmias in this case.

## Conclusion

This case demonstrates how Purkinje-mediated SC-PVCs can give rise to malignant polymorphic ventricular arrhythmias in the technically challenging context of mirror-image dextrocardia. The coexistence of transient QT prolongation and truncating *AKAP9* variants suggests a possible contribution of reduced repolarization reserve, although a definitive channelopathy could not be established. The successful suppression of VF after a stepwise strategy of PDN and targeted focal ablation underscores the importance of early trigger elimination in patients with recurrent Purkinje-mediated VF, particularly when medical therapy is ineffective or limited by concerns about repolarization. This case highlights the need for careful integration of anatomic, electrophysiologic, and genetic information when evaluating patients with episodic QT prolongation and refractory Purkinje-triggered ventricular arrhythmias.

## Disclosures

The authors have no conflicts of interest to disclose.
